# Insight into the pulmonary molecular toxicity of heated tobacco products using human bronchial and alveolar mucosa models at air–liquid interface

**DOI:** 10.1038/s41598-022-20657-y

**Published:** 2022-09-30

**Authors:** Mizanur Rahman, Martin Irmler, Micol Introna, Johannes Beckers, Lena Palmberg, Gunnar Johanson, Swapna Upadhyay, Koustav Ganguly

**Affiliations:** 1 Unit of Integrative Toxicology, Institute of Environmental Medicine, Karolinska Institutet, 171 77 Stockholm, Sweden; 2 Institute of Experimental Genetics, Helmholtz Zentrum München, Deutsches Forschungszentrum für Gesundheit und Umwelt (GmbH), 85764 Neuherberg, Germany; 3 German Center for Diabetes Research (DZD E.V.), 85764 Neuherberg, Germany; 4 Chair of Experimental Genetics, Technical University of Munich, 85354 Freising, Germany

**Keywords:** Risk factors, Respiratory tract diseases

## Abstract

Heated tobacco products (HTP) are novel nicotine delivery products with limited toxicological data. HTP uses heating instead of combustion to generate aerosol (HTP-smoke). Physiologically relevant human bronchial and alveolar lung mucosa models developed at air–liquid interface were exposed to HTP-smoke to assess broad toxicological response (n = 6–7; ISO puffing regimen; compared to sham; non-parametric statistical analysis; significance: p < 0.05). Elevated levels of total cellular reactive oxygen species, stress responsive nuclear factor kappa-B, and DNA damage markers [8-hydroxy-2′-deoxyguanosine, phosphorylated histone H2AX, cleaved poly-(ADP-Ribose) polymerase] were detected in HTP-smoke exposed bronchial and/or alveolar models. RNA sequencing detected differential regulation of 724 genes in the bronchial- and 121 genes in the alveolar model following HTP-smoke exposure (cut off: p ≤ 0.01; fold change: ≥ 2). Common enriched pathways included estrogen biosynthesis, ferroptosis, superoxide radical degradation, xenobiotics, and α-tocopherol degradation. Secreted levels of interleukin (IL)1ꞵ and IL8 increased in the bronchial model whereas in the alveolar model, interferon-γ and IL4 increased and IL13 decreased following HTP-smoke exposure. Increased lipid peroxidation was detected in HTP-smoke exposed bronchial and alveolar models which was inhibited by ferrostatin-1. The findings form a basis to perform independent risk assessment studies on different flavours of HTP using different puffing topography and corresponding chemical characterization.

## Introduction

Heated tobacco products (HTP) are new and emerging tobacco products (first launched in 2014) that allow the user to inhale nicotine by heating the tobacco (350 °C) instead of burning it at high temperature (> 900 °C as in conventional cigarettes)^1–4^. HTP products are currently available in about 50 countries^1–5^. The total sale of HTP products is forecasted to reach nearly 68 billion USD by 2027, a seven-fold increase from 2020^6^. HTP products are increasingly used, especially by the youth (15–24 years), current smokers and former smokers^7–9^. The tobacco industry claims harm reduction by HTP use compared to conventional cigarettes (CC). This remains questionable at the present state of knowledge as long-term toxicity data are missing. The operational technique (heating instead of combustion) for nicotine delivery used in HTPs also enable the tobacco industry to bypass the tobacco directives^10^. Most of the HTP research data currently available were obtained with tobacco heating system 2.2 which has been marketed as “IQOS” (I Quit Ordinary Smoking) by Philip Morris International (PMI)^4, 5^. The IQOS is operated by a battery-powered heating device with a heating blade that is inserted into a HTP specific tobacco unit (heatsticks or HTP sticks) containing processed tobacco leaf^4, 5^. The HTP sticks comes in different flavours (e.g. menthol, nutty, intense tobacco, mint). Upon heating, the stick releases a nicotine containing aerosol (HTP-smoke) that is inhaled by the user. The current scientific literature on HTP is dominated by tobacco industry sponsored research^5, 11, 12^. Till date only few independent studies on HTP have been performed and reports of short-term pathophysiological effects are reported^13^. Therefore, there is an urgent need of independent and comprehensive risk assessment studies on HTPs.

In general, the content of several harmful and potentially harmful constituents (HPHC) including nicotine, tobacco specific nitrosamines, tar, carbon monoxide, hydrogen cyanide, ammonia, phenol, volatile organic compounds, polycyclic aromatic hydrocarbons, aromatic amines, reactive oxygen species (ROS), carbonyls^4, 14–19^ are considerably lower in HTP-smoke compared to CC-smoke. However, some studies reported comparable nicotine and tar content, and percentage of free base in HTP-smoke and CC-smoke^18^. Highly toxic formaldehyde cyanohydrin is detected in HTP-smoke^20^. Almost three times higher levels of potentially carcinogenic acenapthene is also reported in HTP-smoke than in CC-smoke^21^. Evidence of pyrolysis products, propylene glycol and glycerin have been reported in HTP-smoke^18, 19^. Examination of PMI’s IQOS emission data^12^ on the US Food and Drug Administration’s (FDA) HPHC list revealed 56 of 93 constituents to be higher in HTP-smoke compared to CC-smoke. PMI reported levels of only 40 of 93 of these HPHC on FDA list. Among those not reported, fifteen HPHCs were two-fold or more and seven were more than ten-fold higher in HTP-smoke than in CC-smoke^12^. Altogether, these data show that, compared to CC-smokers, HTP-smokers inhale lower amounts of some toxicants but comparable or higher amounts of other toxicants^11^. Some studies have shown that the total particulate matter present in the mainstream HTP-smoke (range: 12.9–55.8 mg/heat stick) to be higher than that of CC-smoke (range: 9.8–37.7 mg/cigarette) and is dependent on the flavour as well as puffing regime^14–19^. The dimensional and volatility characterization of four different flavours of mainstream HTP-smoke by Pacitto et al.^22^ reported the median values of the total particle concentration (volatile and non-volatile) to range between 7.04 × 10^7^ to 9.67 × 10^7^ particles/cm^3^. The corresponding particle number distribution modes were about 100 nm. The volatility analysis further demonstrated that the particle number distribution mode decreases down to approximately 20 nm (at 300 °C)^22^. The study also estimated that the dose received by HTP-smokers in terms of non-volatile amount of particle surface area is 1–2 mm^2^ per puff (10–20 mm^2^ per heat-stick)^22^.

Emerging evidence from clinical studies assessing the effects on smokers and COPD patients switching to HTP are insufficient to draw any conclusions^13, 23–26^. However, reports on HTP use associated acute eosinophilic pneumonia are alarming^27–29^. Inflammatory cell recruitment, cytotoxicity, secretion of pro-inflammatory cytokines, and oxidative stress response have been shown as the primary pulmonary effects of exposure to HTP-smoke^23, 30^. Long-term chronic inhalation exposure of HTP-smoke in female rats resulted in significantly increased lung weight, bronchoalveolar lavage inflammatory cell recruitment, epithelial hyperplasia and metaplasia, and elevated levels of bronchoalveolar lavage inflammatory markers^23, 24^. Short term inhalation exposure to HTP-smoke (Health Canada Intense puffing regimen)^31^ in mice resulted in increased albumin in the bronchoalveolar lavage fluid indicating lung epithelial barrier leakage and infiltration of leukocytes along with increased levels of pro-inflammatory cytokines in the lung^32^. In vitro studies using human bronchial epithelial cells demonstrated that HTP-smoke resulted in oxidative stress and pro-inflammatory response^30, 33^. Higher cytotoxicity was also reported in human bronchial epithelial cells following HTP-smoke exposure compared to corresponding sham^23, 30–33^. Moreover, most of the HTP toxicological data was compared to CC data to showcase harm reduction. However, this does not prove the safety of HTP products, as they may cause severe adverse health effects even if the potency is lower compared to CC-smoke.

The current state of knowledge on HTP-smoke mediated pulmonary toxicity is limited. There is a lack of knowledge on the toxicological response of HTP-smoke exposure in general and particularly from different lung regions such as the conducting (bronchial) and respiratory (alveolar) zones. The cellular architecture and the corresponding molecular signature of the bronchial and alveolar of the lung are distinctly different and therefore the toxicological responses may vary as well in these lung regions. It also needs to be ascertained if and/or how HTP-smoke exposure mediated toxicity is flavour and/or puffing topography (standard or intense) dependent. In this work we hypothesized that there may be common as well as different toxicological response to HTP-smoke exposure in the bronchial and alveolar lung regions.

To address some of the above aspects, we aimed to assess the toxicological response of HTP-smoke (single flavour) using the International Organization for Standardization (ISO) puffing regimen in our established physiologically relevant bronchial (bro) and alveolar (alv) lung mucosa models developed at air–liquid interface (ALI)^34, 35^. The broad toxicological endpoints identified for assessment were (1) oxidative stress, (2) DNA damage, (3) altered transcript expression, and (4) secretion of pro-inflammatory cytokines. Pathway enrichment analysis of the altered gene expression was performed to identify unique as well as common candidate biological mechanisms that may drive HTP-smoke mediated toxicity in the bronchial and alveolar lung regions. The bronchial mucosa models (bro-ALI) were developed using human primary bronchial epithelial cells (PBEC) and the alveolar model (alv-ALI) was developed using representative human type II pneumocytes. Our study provides a comprehensive insight into the molecular pulmonary toxicity of HTP-smoke in two (bronchial and alveolar) different lung regions.

## Methods

**Figure 1:**
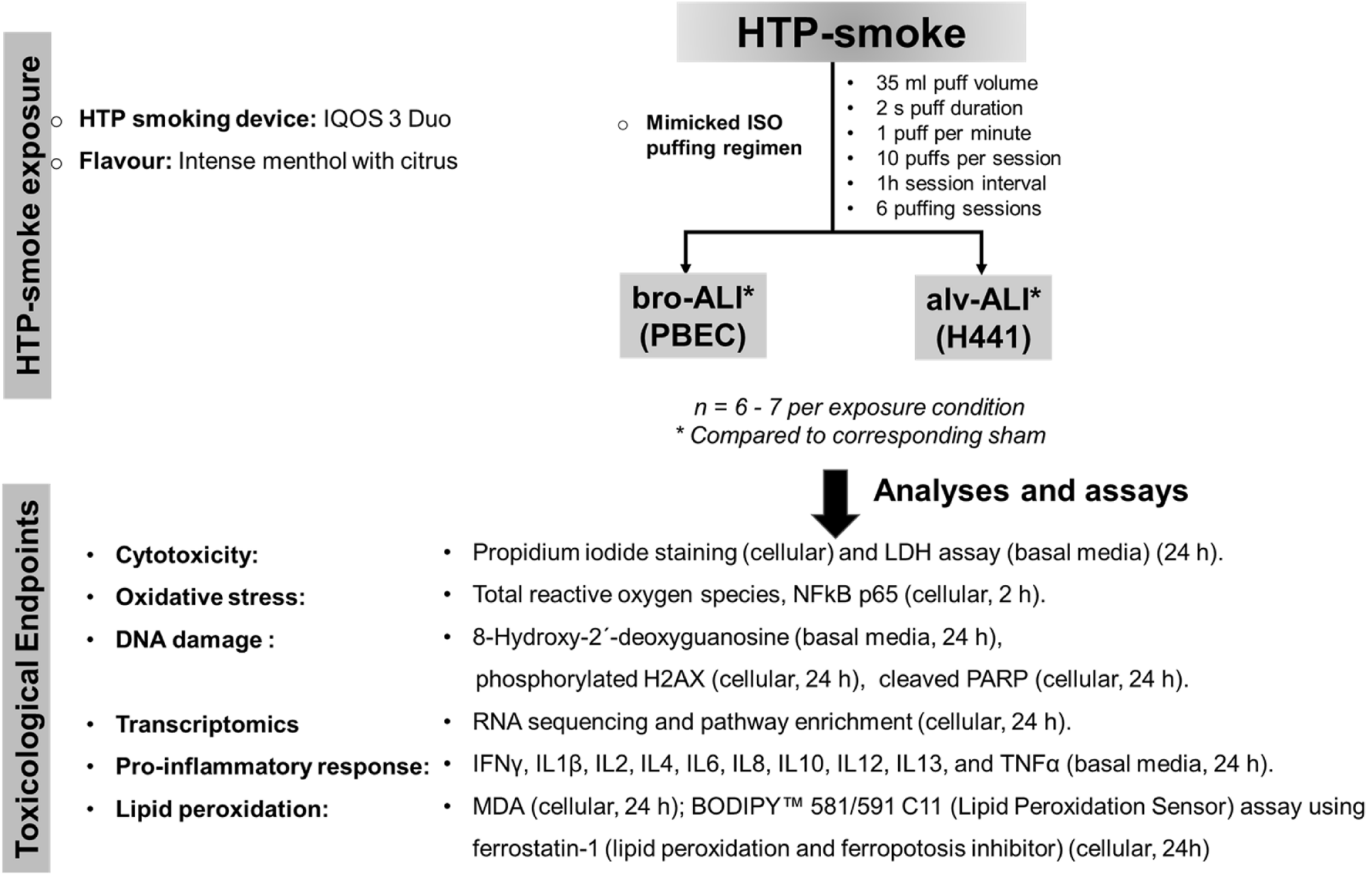
Schematic presentation of the overall experimental design outlining the exposure regimen and endpoints. ALI: air–liquid interface; alv-ALI: alveolar mucosa model at ALI; bro-ALI: bronchial mucosa model at ALI; h: hours; H441: NCI-H441 (ATCC HTB-174) cell line; HTP: heated tobacco product; IFNγ: interferon gamma; IL: interleukin; IQOS: I quit ordinary smoking; LDH: lactate dehydrogenase; MDA: malondialdehyde; NFkB: nuclear factor kappa-light-chain-enhancer of activated B cells; PARP: cleaved poly [ADP-Ribose] polymerase; PBEC: human primary bronchial epithelial cells; TNFα: tumor necrosis factor alpha; γH2AX: phosphorylated histone H2AX.

An overview of the experimental design, HTP-instrument, HTP stick, and puffing regimen used for performing HTP-smoke exposure on bro-ALI and alv-ALI models together with the different analyses and assays is provided in Fig. 1.

### HTP-smoke generation

The HTP-smoke was generated using IQOS 3 Duo HTP instrument and HEETS-Kelly Selection (flavour: intense menthol with citrus) HTP sticks. A one-day exposure experiment consisted of 6 puffing sessions conducted with 1 h intervals. Each session consisted of 10 puffs according to the International Organization for Standardization (ISO) puffing regimen (puff volume: 35 mL, puff duration: 2 s, puff frequency: 1 puff per min)^36^. There was 1 h (h) interval in between each session. Therefore, each bro-ALI and alv-ALI model was exposed to 60 puffs in total.

### HTP-smoke exposure

HTP-smoke exposure experiments were carried out using the exposure system previously illustrated^35^. Nicotine quantification (14.1 ± 1.7 µg/mL; %CV: 11.8) was performed using liquid chromatography-mass spectrometry (LC–MS/MS) analyses to inspect its distribution across all the wells in the 12 well cell culture plate containing 1 mL cell culture media in each well following 1 puffing session (“Supplementary material S1”). An air-tight pre-heated glass syringe was used to repeatedly collect 35 mL of HTP-smoke and inject it in a 3 L desiccator glass jar (37 °C and 60% humidity). The bro-ALI and alv-ALI models were exposed to HTP-smoke or clean air (sham) for 15 min (min) under identical conditions. Thereafter, the lung models were transferred to a cell incubator (37 °C, 60% humidity and 5% CO_2_) for 1 h until next exposure session. At the completion of repeated exposures (i.e. 6 puffing sessions), the lung models were incubated for 24 h prior to collection of basal media and cell inserts. All HTP-exposed samples were compared to their corresponding sham exposed (clean air) samples (n = 6–7 independent experiments per exposure condition). The replicates were randomly distributed in the plates and experiments performed on different days were used for both bro-ALI (developed from one donor; different vials) and alv-ALI (developed from different cell vials) for the different assays.

### Bronchial and alveolar lung mucosal models

#### Bronchial model

The bro-ALI model was developed using PBEC harvested from macroscopically normal bronchial tissue obtained from one donor in connection with lobectomy following written and informed consent. The protocol was approved by the Swedish Ethical Review Authority (Institutional ethic committee reference number: 99–357; approved on 10th January 2000). All methods were performed in accordance with the relevant guidelines and regulations. The detailed protocol and details of cellular differentiation (Club cells, goblet cells, basal cells, ciliated cells, etc.) of the bro-ALI model has been described previously and the model has been in several studies^34, 35, 37–41^.

#### Alveolar model

The alv-ALI model was developed using NCI-H441 (ATCC HTB-174; derived from the pericardial fluid of a patient with papillary adenocarcinoma of the lung) cell line and the detailed protocol and model characteristics have been described recently^35, 37^. The NCI-H441 cells, representative of human type II pneumocytes, express constitutively the mRNA and protein of the major surfactant apo-protein. NCI-H441 cells were co-cultured with HULEC-5a (ATCC CRL-3244) representative of human lung microvascular endothelial cells for this purpose. The alv-ALI model characterization included light microscopy, confocal microscopy, transmission electron microscopy, and transepithelial electrical resistance measurement^35, 37^. Morphological characterization of the alv-ALI model demonstrated the presence of tight junction protein 1, lamellar bodies, surfactant protein C, microvilli, lipid bodies, desmosome, and tight junctions^35, 37^.

### Assessment of HTP-smoke related pulmonary response

#### Cytotoxicity assessment

Membrane integrity-based cell viability assays were used for cytotoxicity assessment of bro-ALI (n = 6) and alv-ALI (n = 6) models following 24 h after the HTP-smoke exposure. Colorimetric lactate dehydrogenase (LDH; Thermo Fisher scientific Rockford, lL, US, catalog # 88953) assay and propidium iodide (PI; BD bioscience, San Jose, CA, US, catalog # 556463) staining were used according to manufacturer’s instruction and as described previously^35, 37^. The LDH assay was measured using BioTek 800 TS absorbance reader (Santa Clara, CA, US) and PI assay was performed using flow cytometry (BD LSRFortessa cell analyzer, BD bioscience, San Jose, CA, US). The flow cytometric data was analyzed using FlowJo software-7.6.1 (BD bioscience, San Jose, CA, US). Data are presented as percentage positive PI cells and interquartile ranges. LDH assay is shown as medians of absorbance (450 nm) and interquartile ranges. The same instruments and software were used for all subsequent enzyme linked immune sorbent assays (ELISA) and flow cytometric analysis mentioned hereafter.

### Total ROS and nuclear factor kappa-light-chain-enhancer of activated B cells (NFkB)

Total cellular ROS and NFkB was measured in both bro-ALI (n = 6) and alv-ALI (n = 6) following 2 h post HTP-smoke exposure using flow cytometry according to manufacturer’s instruction. Cells were stained with CellROX green reagent (ThermoFisher Scientific, Rockford, lL, US, Catalog #: C10444) for 30 min in incubator. Following incubation, cells were washed, collected and ROS were measured as previously described^35^. Expression of the stress responsive transcription factor NFkB was assessed following staining of the cells using NFkB p65 subunit kit according to manufacturer’s instruction (BD biosciences, San Jose, CA, US, catalog # 560335). Shortly, cells were stained with PE-Cy7 conjugated anti-NFkB p65 for 30 min, followed by washing, and measurement by flow cytometry. Mean fluorescent intensity (MFI) represents the level of total ROS and NFkB.

### Assessment of DNA damage

DNA damage was assessed by measuring the concentration of 8-hydroxy-2′-deoxyguanosine (8-OHdG) in the basal media of HTP-smoke exposed bro-ALI (n = 6) and alv-ALI (n = 6) using a commercially available competitive enzyme linked immune sorbent assay (ELISA) kit (ThermoFisher Scientific; Rockford, lL, US, catalog # EIADNAD) according to manufacturer’s instructions. Further, cellular phosphorylated histone H2AX (γH2AX) and cleaved Poly [ADP-Ribose] Polymerase (PARP) levels as markers of DNA damage and repair were also assessed by flow cytometry in HTP-exposed bro-ALI and alv-ALI models 24 h post-exposure according to manufacturer’s instruction by flow cytometry. The cells were stained with allophycocyanin (APC) conjugated anti- γH2AX and PE conjugated anti-PARP antibodies for 30 min, and fluorescence intensity measured as described above by flow cytometry.

### Transcriptomic analysis

Transcriptomic analysis was performed using the UPX 3′ RNA sequencing technology (RNAseq; Qiagen Genomic Services, Hilden, Germany) as recently described^37^. To determine the differentially expressed genes following HTP-smoke exposure in both bro-ALI (n = 7) and alv-ALI (n = 6), cells were collected in Qiagen RLT buffer 24 h post exposure (Qiagen, Hilden, Germany, catalog # 74104), snap frozen, and dispatched in dry ice to the service laboratory according to the service provider’s instructions. For alv-ALI, the apical layer containing type II pneumocytes were collected. A raw p value ≤ 0.01 and fold change ≥ 2 was set to select the differentially expressed genes. Gene symbols were obtained from Ensembl (Gene2ensembl; BioMart) and NCBI (Homo_sapiens.gene_info.gz). Heatmaps showing the top 25 upregulated and 25 down regulated genes (by fold change) were generated in R^42^. Ensembl genes without gene symbol annotation were omitted from heatmaps. RNAseq data is deposited at the Gene Expression Omnibus database at NCBI [GSE198082; https://www.ncbi.nlm.nih.gov/geo/; accessed on 14th March 2022].

### Pathway and enrichment analysis

For the biological interpretation of the differentially regulated genes, canonical pathway and upstream regulator analyses were performed using the QIAGEN’s Ingenuity Pathway Analysis software (IPA, QIAGEN Redwood City, www.qiagen.com/ingenuity, content version 70750971, Release Date 2021-10-22). Significant terms were selected using Fisher`s Exact Test p-values (p < 0.05 for pathways and p < 0.01 for upstream regulators), z-scores ≥ 2 indicate activation, and z-scores ≤ − 2 indicate inhibition.

### Secreted cytokine concentration

Concentrations of pro-inflammatory cytokines interferon gamma (IFNγ), interleukin (IL) 1β, IL2, IL4, IL6, IL8, IL10, IL12, IL13, and tumor necrosis factor alpha (TNFα) were measured in the basal media of the bro-ALI (n = 6) and alv-ALI (n = 6) following 24 h post-exposure as described previously^35, 37^. IL8 was measured using ELISA (R & D Systems, Minneapolis, MN, US, Catalog # DY208) and the remaining cytokines were measured using the V-plex immunoassay platform of Meso Scale Discovery Inc (Rockville, MD, US) at the Clinical Biomarkers facility, Science for Life Laboratory, Uppsala University, Sweden. Basal media of the samples used for RNAseq analysis were used for protein secretion analysis.

Quantitative real time polymerase chain reaction (qRT-PCR) was performed for selected pro-inflammatory genes (*IFNG*, *IL1B*, *IL4*, *IL8*, and *IL13*) showing significantly different secreted protein levels in the basal medium to additionally assess their transcript expression as previously described^35^. Actin beta (*ACTB*) was used as the reference control.

### Lipid peroxidation

Concentration of malondialdehyde (MDA) was measured to assess lipid peroxidation^43^ using cell lysate of HTP-smoke exposed bro-ALI (n = 6) and alv-ALI (n = 6) models 24 h post-exposure using a commercially available colorimetric assay and according to manufacturer’s instruction. (Sigma Aldrich, St Lois, MO, US, catalog # MAK085). Further, BODIPY™ 581/591 C11 (Lipid Peroxidation Sensor) (ThermoFisher Scientific; Rockford, lL, US, Catalog #: D3861) was used to assess lipid peroxidation^44^ in HTP-exposed bro-ALI and alv-ALI 24 h post exposure using flow cytometry. Ferrostatin-1 (10 nM as working solution; Sigma Aldrich St Lois, MO, US, catalog # SML0583) was used as an inhibitor lipid peroxidation in this assay which in turn also acts as a ferroptosis inhibitor^44, 45^ The cells were incubated with 2 µM BODIPY for 30 min, washed, and fluorescence intensity was measured by flow cytometry as described above.

### Statistics

The results (flow cytometry, protein concentration, ELISA, qRT-PCR) were expressed as medians and interquartile ranges (25th–75th percentiles). Both bro-ALI and alv-ALI models are well-differentiated tissue-like models, which contain multiple layers of cells including different cell types of unique distribution. Hence, in this study, every model (ie. both bro-ALI and alv-ALI) are considered as a unique in vivo-like in vitro model with its own distribution of different cell types and the number of cells present might differ. Therefore, differences between treatments were examined by non-parametric statistical analysis (Wilcoxon signed rank test or Friedman test followed by Wilcoxon signed rank test, as appropriate) using GraphPad Prism (9.3.1) software (LaJolla, CA, US). A p value < 0.05 was considered as significant. The statistic method used in the RNA sequencing and pathway analysis are described in the respective sections.

### Ethical approval

Institutional Review Board Statement: All procedures performed for the in vitro study were in accordance with the approval of the Swedish Ethical Review Authority (Institutional ethic committee reference number 99-357; Dated: 10.01. 2000.).

### Informed consent statement

All subjects were enrolled after informed consent process for creation of the primary cell bank.

## Results

### Cytotoxicity

The exposure regime used in this study was not cytotoxic (> 87% cell viability) in the bro-ALI and alv-ALI models as indicated by PI staining and LDH assays (Supplementary Fig. S1). More specifically, we saw in case of both bro-ALI (0.4%; p = 0.03) and alv-ALI (7.6%; p = 0.03) a slight increase of PI positive cells following HTP-smoke exposure compared to sham. Released LDH levels were not different among HTP exposed bro-ALI compared to sham. In case of alv-ALI, released LDH levels were increased (p = 0.03) consistent to PI staining in the HTP exposed samples compared to sham. Therefore, the described exposure regime was suitable to proceed with all further experiments.

### Oxidative stress

**Figure 2:**
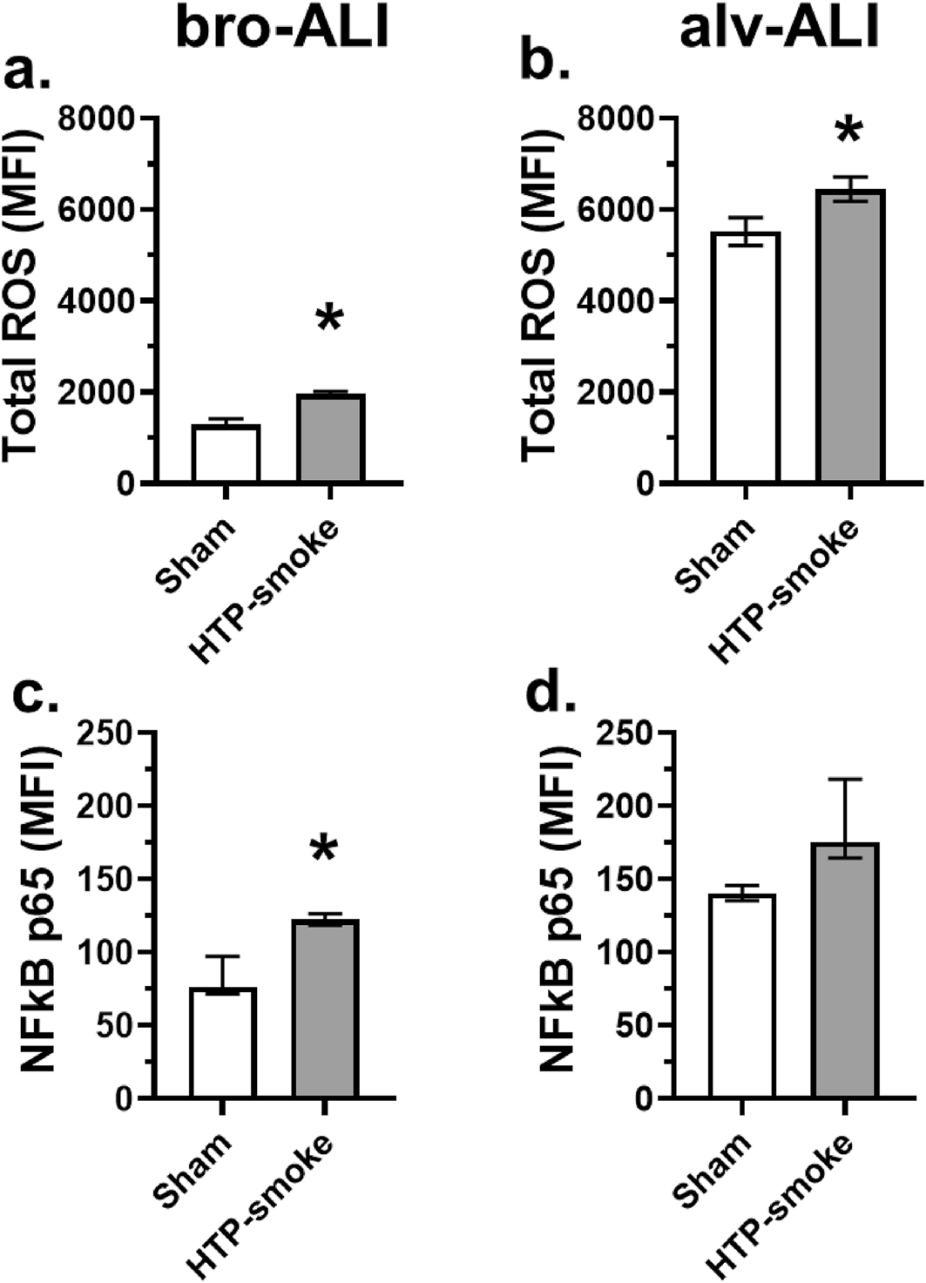
Assessment of oxidative stress response by measurement of (**a**, **b**) total cellular reactive oxygen species (ROS) and (**c**, **d**) expression of nuclear factor kappa-light-chain-enhancer of activated B cells (NFkB) p65 subunit by flow cytometry in sham exposed and HTP-smoke exposed bro-ALI and alv-ALI models. Data are shown as medians and interquartile ranges. n = 6 per exposure condition; non-parametric statistical analysis (Wilcoxon signed rank test), *p < 0.05. ALI: air–liquid interface; alv-ALI: alveolar mucosa model at ALI; bro-ALI: bronchial mucosa model at ALI; HTP: heated tobacco product, MFI: mean fluorescent intensity.

Increased total cellular ROS was detected in both bro-ALI (51%; p = 0.03; Fig. 2a) and alv-ALI (17%; p = 0.03; Fig. 2b) following HTP exposure compared to corresponding sham. as were the levels of NFkB p65 subunit in bro-ALI (61%; p = 0.03; Fig. 2c) and alv-ALI (25%; p = 06; Fig. 2d) following HTP-smoke exposure compared to sham.

### DNA damage

**Figure 3:**
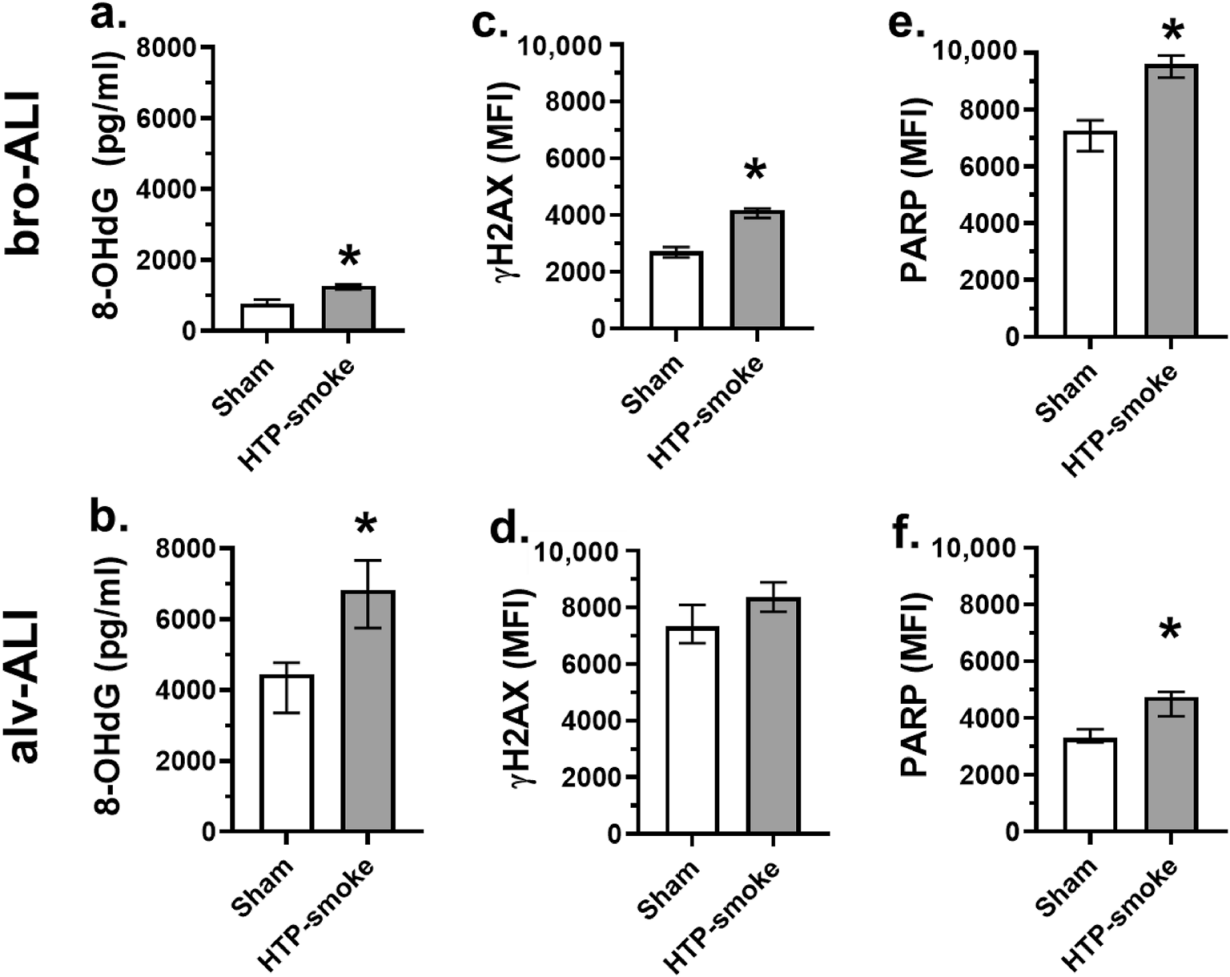
Assessment of the levels of DNA damage markers (**a**, **b**) 8-hydroxy-2′-deoxyguanosine (8-OHdG; pg/mL), (**c**, **d**) cellular phosphorylated histone H2AX (γH2AX) and (**e**, **f**) cleaved poly [ADP-Ribose] polymerase (PARP) levels in sham exposed and HTP-smoke exposed bro-ALI and alv-ALI models. Data are shown as medians and interquartile ranges. n = 6 per exposure condition; non-parametric statistical analysis (Wilcoxon signed rank test), *p < 0.05. ALI: air–liquid interface; alv-ALI: alveolar mucosa model at ALI; bro-ALI: bronchial mucosa model at ALI; HTP: heated tobacco product; MFI: mean fluorescent intensity.

The DNA damage marker 8OH-dG was significantly increased in the basal media of both models (bro-ALI: 65%; p = 0.03; Fig. 3a; and alv-ALI: 35%; p = 0.03; Fig. 3b) following HTP-smoke exposure compared to corresponding sham. The cellular expression of γH2AX was significantly increased in bro-ALI (53%; p = 0.03; Fig. 3c) but not in alv-ALI (p = 0.09; Fig. 3d) following HTP exposure. Cleaved PARP levels were significantly increased in both bro-ALI (33%; p = 0.03; Fig. 3e) and alv-ALI (44%; p = 0.03; Fig. 3f) following HTP-smoke exposure.

### Transcriptomic alterations and proinflammatory cytokine secretion

#### Bronchial model

**Figure 4:**
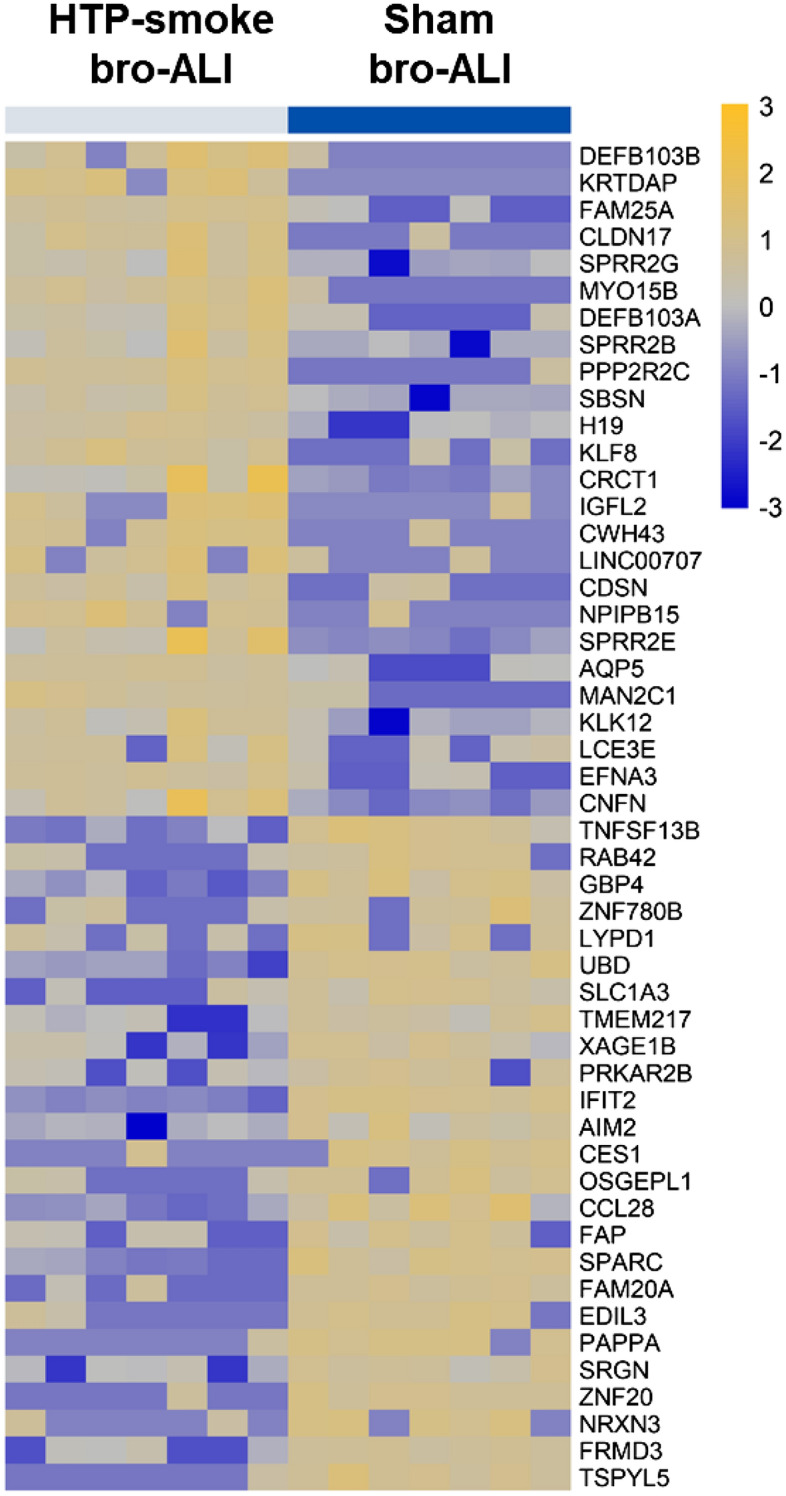
Heatmap of the top 25 up regulated and 25 down regulated genes in the bro-ALI model following exposure to HTP-smoke. n = 7 per exposure condition; significantly (raw p < 0.01) regulated genes with the highest fold changes are shown. Genes were ordered by fold-change (HTP-smoke vs Sham) and relative gene expression values are shown across samples (z-scales to mean expression per row). A complete list of the 724 differentially regulated genes is provided in Supplementary Table S1. bro-ALI: bronchial mucosa model at air–liquid interface; HTP: heated tobacco product.

**Figure 5:**
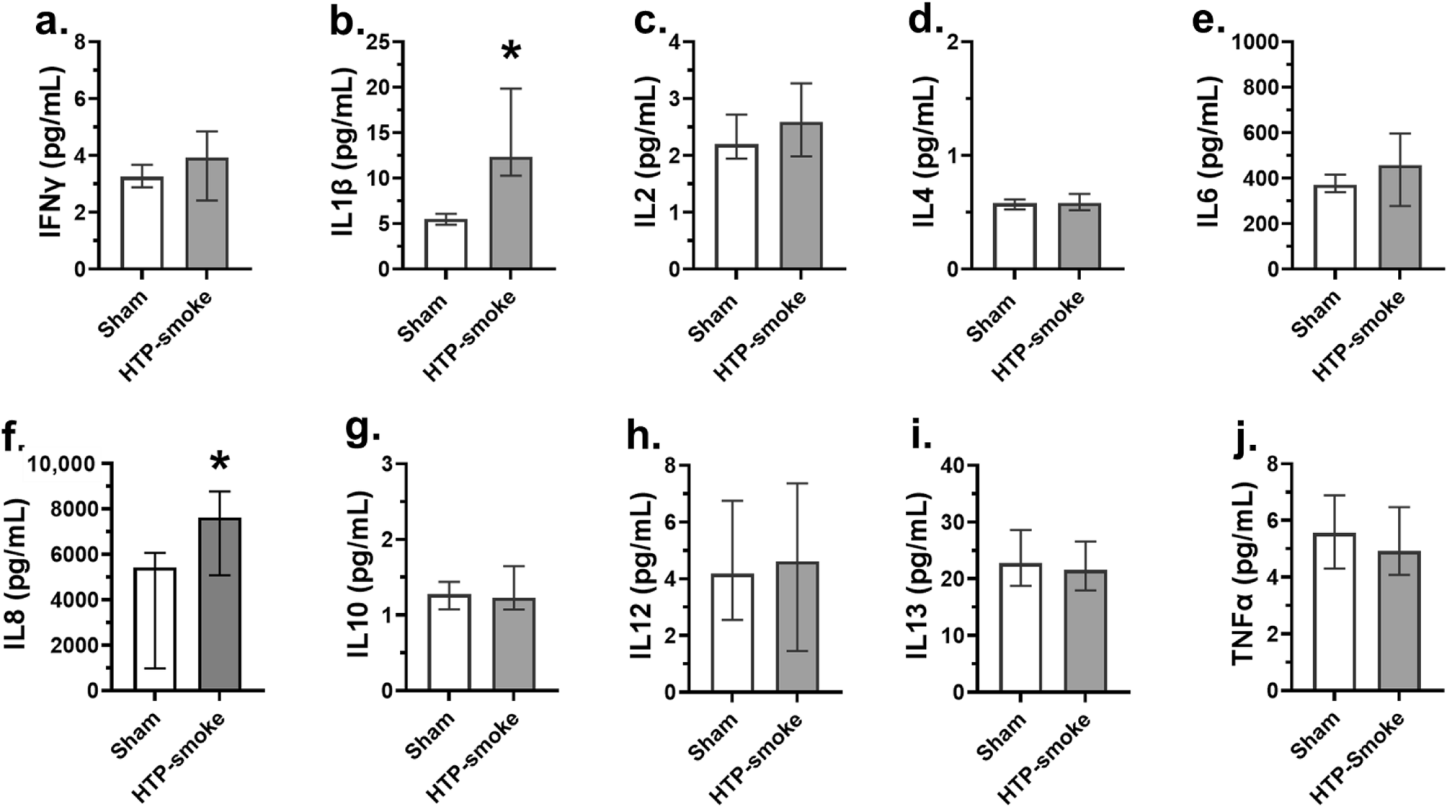
Concentration of secreted proinflammatory cytokines in the basal media of sham exposed and HTP-smoke exposed bro-ALI model. IFNγ: interferon gamma; IL: interleukin; TNFα: tumor necrosis factor alpha. Data are shown as medians and interquartile ranges. n = 6 per exposure condition; non-parametric statistical analysis (Wilcoxon signed rank test); *Significance: p < 0.05. bro-ALI: bronchial mucosa model at air–liquid interface; HTP: heated tobacco product.

A total of 724 genes were differentially regulated (424 upregulated and 300 down regulated; n = 7; p ≤ 0.01; fold change ≥ 2) in the bro-ALI model post 24 h exposure to HTP-smoke compared to sham (Supplementary Table S1). Figure 4 shows a heat map of the top 25 upregulated and 25 down regulated genes. Enrichment analysis using the 724 differentially regulated genes identified 62 significantly enriched canonical pathways (Supplementary Table S2). Selected enriched canonical pathways are given in Table 1 and include interferon signaling, liver X receptor (LXR)/retinoid X receptor (RXR) activation, glucocorticoid receptor signaling, p38 mitogen-activated protein kinase (MAPK) signaling, IL13 signaling pathway, Toll like receptor signaling, peroxisome proliferator-activated receptors (PPAR) signaling, aryl hydrocarbon receptor (AHR) signaling, IL17 signaling, and airway pathology in chronic obstructive pulmonary disease (COPD). The secreted levels of proinflammatory cytokines (Fig. 5a–j) IL1ꞵ (p < 0.0001; Fig. 5b) and IL8 (p = 0.04; Fig. 5f) were increased in bro-ALI model (n = 6). None of the other examined cytokines was significantly altered by exposure to HTP-smoke in the bro-ALI model (Fig. 5).

**Table 1:** Shown are selected enriched canonical pathways (Fisher’s Exact test p-value < 0.05) associated with the sets of differentially regulated genes in the bronchial (724 genes) and alveolar (121 genes) mucosa models developed at air liquid interface (bro-ALI and alv-ALI) post 24 h exposure to heated tobacco product (HTP)-smoke compared to sham. Terms observed with both datasets are italicized.

Canonical pathways	p-value	Activation	Molecules
**Bronchial mucosa model** (bro-ALI)			
Interferon signaling	5.25E − 06	Inhibited	*IFI35, IFIT1, IFIT3, IFITM2, IRF1, ISG15, MX1, STAT2*
LXR/RXR activation	8.51E − 06	Inhibited	*ABCG1, APOD, APOL1, IL1R1, IL1RN, IL33, IL36RN, PON3, S100A8, SAA1, SAA2, SAA4, TF, TLR3*
Glucocorticoid receptor signaling	2.14E − 04	–	*CDKN1A, CEBPA, DUSP1, GABPB2, HLA-B, HLA-C, HLA-DPA1, HLA-F, IL18R1, IL1R1, IL1RN, KRT16, KRT40, KRT6A, KRT6B, KRT6C, KRT78, KRT80, MMP13, MT-ATP6, MT-ND2, MT-ND4L, MT-ND5, MT-ND6, PIK3R1, RARB, SERPINE1, SHC1, TAF15, TGFB3, TGFBR2, VIPR1*
*Ferroptosis signaling pathway*	1.17E − 03	–	*ALOX15B, ALOXE3, ANGPTL4, CDKN1A, CHAC1, FTL, H2BC5, NOX1, SLC7A11, TF, TXNRD1*
p38 MAPK signaling	1.95E − 03	–	*CREB3L1, DUSP1, IL1R1, IL1RN, IL33, IL36RN, IRAK3, MKNK2, TGFB3, TGFBR2*
IL-13 signaling pathway	5.13E − 03	–	*ALOX15B, CXCL6, DEFB103A/DEFB103B, DEFB4A/DEFB4B, DUSP1, HBEGF, IL33, PIK3R1, TGFB3*
Toll-like receptor signaling	6.03E − 03	–	*IL1RN, IL33, IL36RN, IRAK3, TICAM1, TLR3, UBD*
PPAR signaling	1.00E − 02	–	*CITED2, IL1R1, IL1RN, IL33, IL36RN, PDGFB, PPARD, SHC1*
Aryl hydrocarbon receptor signaling	1.38E − 02	–	*ALDH3A1, ALDH3B2, CCNA1, CDKN1A, CYP1A1, CYP1B1, NCOA7, NQO1, RARB, TGFB3*
IL-17 signaling	1.58E − 02	Inhibited	*CLCF1, CXCL5, DEFB103A/DEFB103B, DEFB4A/DEFB4B, IL33, MMP13, PIK3R1, TGFB3, TNFSF10, TNFSF13B, TNFSF15*
*Superoxide radicals degradation*	1.95E − 02	–	*CYGB, NQO1*
*α-Tocopherol degradation*	3.02E − 02	–	*CYP4F11, CYP4F3*
*Xenobiotic metabolism signaling*	3.31E − 02	–	*ALDH3A1, ALDH3B2, CES1, CES2, CHST11, CITED2, CYP1A1, CYP1B1, FTL, NQO1, PIK3R1, PPP2R2C, SULT1E1, SULT2B1*
*Estrogen biosynthesis*	3.63E − 02	–	*AKR1B10, AKR1C1/AKR1C2, CYP1A1, CYP1B1*
Airway pathology in chronic obstructive pulmonary disease (COPD)	4.68E − 02	–	*APOD, CLCF1, IL33, TGFB3, TNFSF10, TNFSF13B, TNFSF15*
**Alveolar mucosa model (alv-ALI)**			
*Estrogen biosynthesis*	4.17E − 05	–	*AKR1C1/AKR1C2, AKR1C3, CYP1B1, HSD17B7*
Glutathione biosynthesis	5.50E − 05	–	*GCLC, GCLM*
*Ferroptosis signaling pathway*	2.51E − 03	–	*FTH1, FTL, GCLC, H2AC20*
Bile acid biosynthesis, neutral pathway	2.63E − 03	–	*AKR1C1/AKR1C2, AKR1C3*
NRF2-mediated oxidative stress response	3.55E − 03	Activated	*FTH1, FTL, GCLC, GCLM, NQO1*
Retinoate biosynthesis I	1.41E − 02	–	*AKR1C1/AKR1C2, AKR1C3*
Xenobiotic metabolism general signaling pathway	2.34E − 02	–	*FTL, GCLC, NQO1*
*Superoxide radicals degradation*	3.39E − 02	–	*NQO1*
*Xenobiotic metabolism signaling*	3.63E − 02	–	*CYP1B1, FTL, GCLC, NQO1*
*α-Tocopherol degradation*	4.17E − 02	–	*CYP4F3*

#### Alveolar model

**Figure 6:**
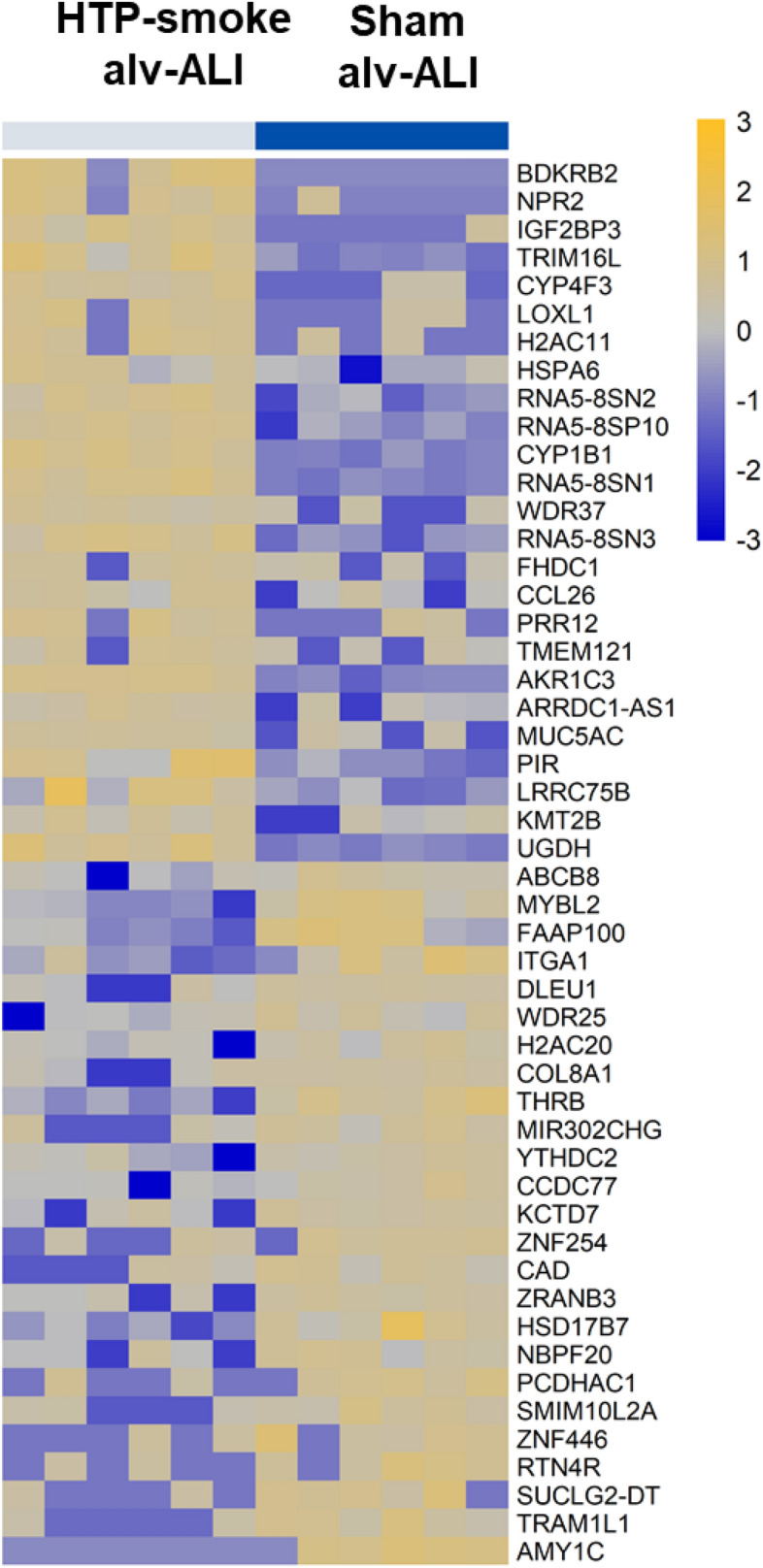
Heatmap of the top 25 up regulated and 25 down regulated genes in the alv-ALI model following exposure to HTP-smoke. n = 6 per exposure condition; significantly (raw p < 0.01) regulated genes with the highest fold changes are shown Genes were ordered by fold-change (HTP-smoke vs Sham) and relative gene expression values are shown across samples (z-scales to mean expression per row). A complete list of the 121 differentially regulated genes is provided in Supplementary Table S3. alv-ALI: alveolar mucosa model at air–liquid interface; HTP: heated tobacco product.

**Figure 7:**
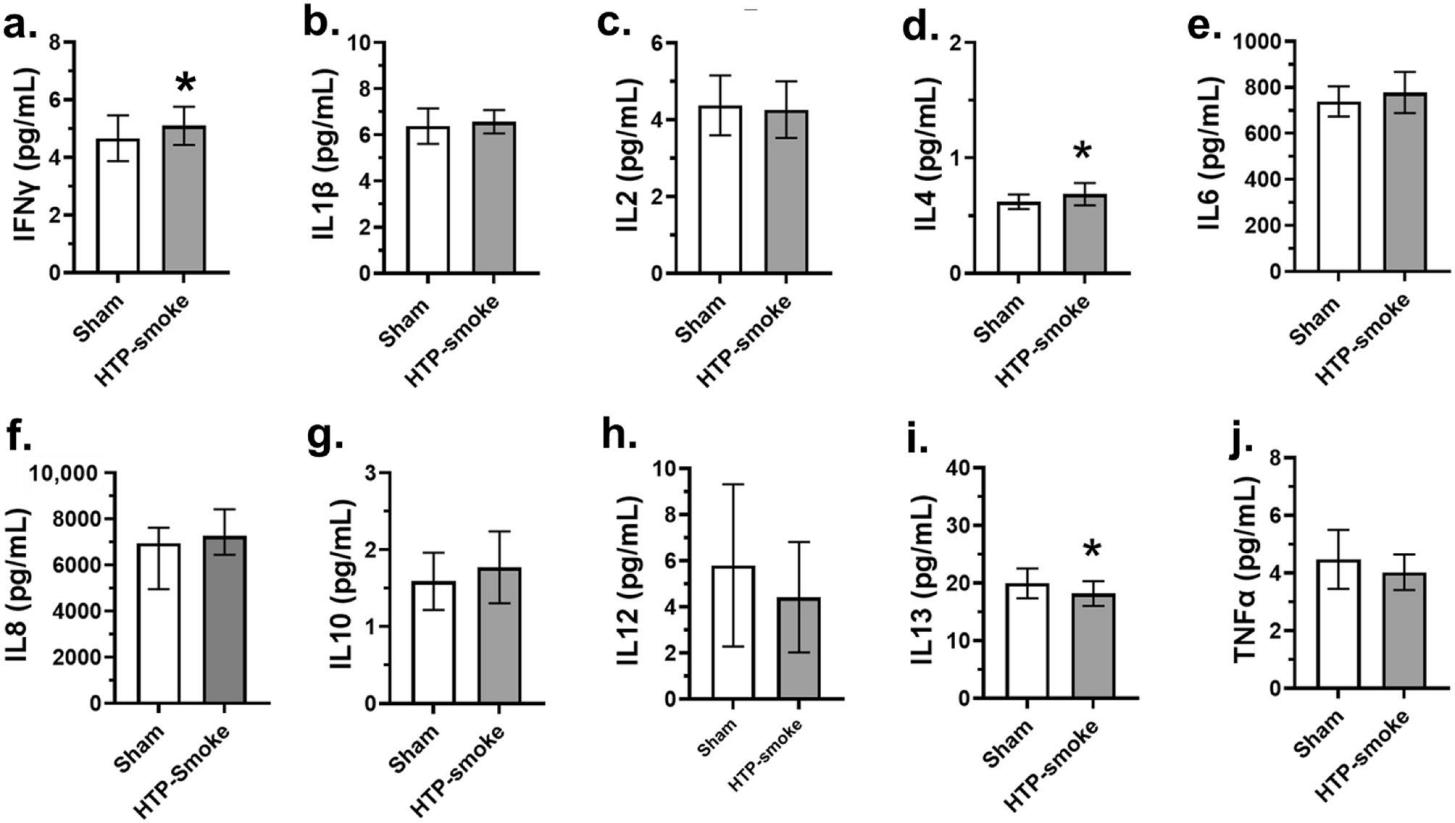
Concentration of secreted proinflammatory cytokines in the basal media of sham exposed and HTP-smoke exposed alv-ALI model. IFNγ: interferon gamma; IL: interleukin; TNFα: tumor necrosis factor alpha. Data are shown as medians and interquartile ranges. n = 6 per exposure condition; non-parametric statistical analysis (Wilcoxon signed rank test); *Significance: p < 0.05. alv-ALI: alveolar mucosa model at air–liquid interface; HTP: heated tobacco product.

A total of 121 genes were differentially regulated (60 upregulated and 61 down regulated; n = 6; p < 0.01; fold change ≥ 2) in the alv-ALI model 24 h after exposure to HTP-smoke compared to sham (Supplementary Table S3). Figure 6 shows a heat map of the top 25 upregulated and 25 down regulated genes. Enrichment analysis using the 121 differentially regulated genes identified 21 significantly enriched canonical pathways (Supplementary Table S4). Selected enriched canonical pathways are provided in Table 1 and include glutathione biosynthesis, bile acid biosynthesis-neutral pathway, nuclear factor erythroid 2-related factor 2 (NRF2) mediated oxidative stress response, retinoate biosynthesis I, and xenobiotic metabolism general signaling pathway. The secretion of of pro-inflammatory cytokines (Fig. 7a–j) IFNγ (p < 0.0001; Fig. 7a) and IL4 (p = 0.01; Fig. 7d) increased whereas that of IL13 decreased (p = 0.02; Fig. 7i) following HTP-smoke exposure.

Consistent with the findings of RNAseq analysis, transcript expression of *IFNG*, *IL1B*, *IL4*, *IL8*, and *IL13* as detected by qRT-PCR remained unaltered in both bro-ALI and alv-ALI exposed to HTP-smoke compared to the corresponding sham (Supplementary Fig. S2).

#### Bronchial and alveolar models

Significantly enriched pathways common to both bro-ALI and alv-ALI included estrogen biosynthesis, ferroptosis signaling pathway, superoxide radicals degradation, xenobiotic metabolism signaling, and α-tocopherol degradation (Table 1). Upstream regulator analysis in bro-ALI predicted the majority of the cytokines (24 out of 28) to be inhibited (Supplementary Table S5). As for example, IL1RN, IL4, IL5, and IL10 were predicted to be activated in bro-ALI but only TNFα for alv-ALI (Supplementary Table S6).

### Lipid peroxidation

**Figure 8:**
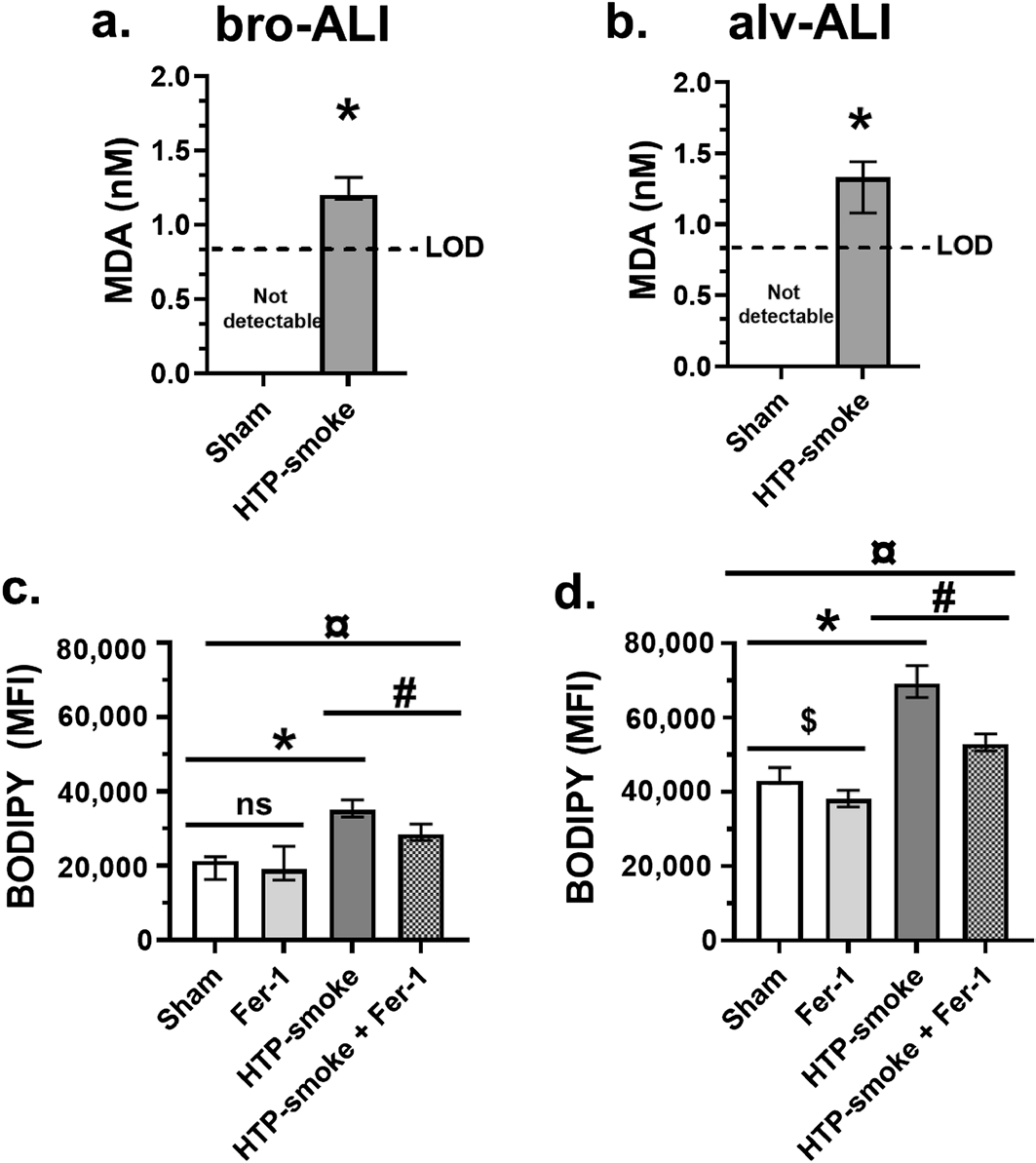
Assessment of lipid peroxidation levels in the bro-ALI and alv-ALI models due to sham exposure and HTP-smoke exposure. (**a**, **b**) Colorimetric malondialdehyde (MDA) assay and (**c**, **d**) BODIPY 581/591 C11 (Lipid Peroxidation Sensor) assay using ferrostatin-1 by flow cytometry. Data are shown as medians and interquartile ranges. n = 6 per exposure condition; non-parametric statistical analysis (Wilcoxon signed rank test or Friedman test followed by Wilcoxon signed rank test, as appropriate); ns: not significant; ^#,¤,$,^*p < 0.05. MDA levels were below the limit of detection (LOD) in both bro-ALI and alv-ALI sham samples. Therefore, a value just below the LOD (0.85 nM) of MDA is assigned to all the sham samples for statistical analysis. ALI: air–liquid interface; alv-ALI: alveolar mucosa model at ALI; bro-ALI: bronchial mucosa model at ALI; Fer-1: ferrostatin-1; HTP: heated tobacco product; MFI: mean fluorescent intensity.

Significantly increased MDA levels were detected in both models (bro-ALI: p = 0.03; Fig. 8a; and alv-ALI: p = 0.03; Fig. 8b) following HTP exposure. MDA levels were undetectable in the sham exposed bro-ALI and alv-ALI models. The lipid peroxidation assay revealed significant increase in both models (bro-ALI: 64%; p = 0.03; Fig. 8c; and alv-ALI: 61%; p = 0.03; Fig. 8d) after HTP-smoke exposure. Addition of ferrostatin-1 significantly reduced the lipid peroxidation in both HTP-exposed models (bro-ALI: − 18%; p = 0.03; and alv-ALI: − 24%; p = 0.03) compared ferrostatin-1 untreated samples. The lipid peroxidation was also significantly reduced by ferrostatin-1 treatment sham samples in alv-ALI model (− 12%; p = 0.03) but not in the bro-ALI model. Although reduced by ferrostatin-1, the lipid peroxidation levels after HTP-smoke exposure remained higher in both models (bro-ALI: (34%; p = 0.03) and alv-ALI (23%; p < 0.03) compared to sham.

## Discussion

To comprehensively assess the adverse effects of HTP-smoke, we exposed physiologically relevant human bronchial and alveolar lung mucosa models at ALI to HTP-smoke using an ISO puffing regimen. The bro-ALI and alv-ALI models represents the conducting and respiratory zones of the lung respectively thereby allowing us to assess the adverse effects of HTP exposure at two different levels of the lung. The conducting and respiratory zones of the lung have their unique cellular architecture and molecular signature many of which are present in the physiologically relevant bronchial and alveolar models used in this study and mentioned earlier. Increased oxidative stress due to HTP-smoke exposure in both bro-ALI and alv-ALI was detected by elevated cellular total ROS and NFkB levels. Pathways involved in oxidative stress or down-stream of oxidative stress are enriched in the transcriptomic profiles of bronchial and alveolar models exposed to HTP-smoke. This indicates oxidative stress as the main upstream event to HTP-smoke exposure in the bronchial and alveolar models. This is consistent with other studies on HTP-smoke which demonstrated HTP-smoke exposure resulted in oxidative stress^23, 30, 32, 33^. Similar effects have also been widely reported in case of CC-smoke^46–48^. However, the extent of adverse effects was in general lower in case of HTP-smoke compared to CC-smoke^23, 30, 32, 33^. DNA damage on bro-ALI and alv-ALI are indicated by increased levels of 8-OH-dG, γH2AX, and cleaved PARP levels upon HTP-smoke exposure. This is also consistent with reported effects of CC-smoke exposure on oxidative DNA damage^49–52^. Elevated levels of 8-OH-dG, γH2AX, and cleaved PARP levels have been reported in case of CC-smoke exposure, COPD, and lung cancer^43, 53^. 8-OH-dG is a widely studied DNA damage marker^43, 53^. γH2AX functions as a coordinator of DNA damage response by recruiting specific proteins and providing a binding site for the downstream signaling molecules^54^. Several studies have linked exposure to CC-smoke and γ-H2AX formation in vitro and in vivo^54, 55^. PARPs are multidomain proteins that are DNA repair factors which are activated to repair DNA lesions^56^. PARPs are also involved in cellular stress response as well as maintaining cellular homeostasis^56^. Therefore, the findings of this study indicate oxidative DNA damage in both bronchial and alveolar models as another adverse consequence of HTP-smoke exposure.

While interpreting the response of the bronchial and alveolar model to HTP-smoke exposure, it is important to consider the intrinsic characteristics of the two models. First, the bronchial model is developed from human primary bronchial epithelial cells collected from macroscopically normal bronchial tissue whereas the alveolar model is developed from immortalized lung adenocarcinoma cell line exhibiting characteristics of type II pneumocytes. Thus, there are inherent differences between the two cell types used. This is also supported by the higher basal levels of LDH, % PI positive cells, ROS, NFkB, 8-OHdG, γH2AX, and BODIPY in the alveolar model compared to the bronchial model. Further, the bronchial model differentiates into Club cells, goblet cells, basal cells, ciliated cells etc. and each have their unique cellular and molecular characteristics. On the other hand, presence of tight junction protein 1, lamellar bodies, surfactant protein C, microvilli, lipid bodies, desmosome, and tight junctions are features of the alveolar model. The role of the surfactant layer serves as a protective physical barrier in alveoli has been also demonstrated in cell culture^57^. The above reviewed information highlights the fact that each lung model has its own characteristics. Therefore, in this study the data generated from the two models have been dealt separately However, while performing pathway enrichment analysis for biological relevance of the toxicological response, few common enriched pathways (ferroptosis, superoxide radical degradation, xenobiotic metabolism, estrogen biosynthesis, α-tocopherol) were detected among bronchial and alveolar models. Identification of common pathways may provide important clue regarding common toxicological mechanisms at both lung regions apart from lung region specific effects. Based on the finding of ferroptosis as a common enriched pathway, lipid peroxidation assay using ferrostatin-1 inhibitor was performed to identify a plausible biochemical mechanism of HTP-smoke toxicity. Therefore, ferroptosis as one of the important candidate toxicological pathway of HTP-smoke exposure warrant detailed mechanistic evaluation.

In the following section we have briefly addressed some of the enriched canonical pathways in bro-ALI and alv-ALI that are mentioned in Table 1 to outline the plausible mechanisms of HTP-smoke exposure related pulmonary toxicity in each lung region. It is important to note that Supplementary Tables S2 and Tables S4 includes several other pathways which contributes to the toxicity of HTP-smoke specifically for the bronchial and alveolar regions. Enrichment of AHR signaling pathway, glutathione biosynthesis, NRF2-mediated oxidative stress response, superoxide radicals degradation, xenobiotic metabolism signaling following transcriptomic analysis in bro-ALI and/ or alv-ALI in our exposure experiments are consistent with the CC-smoke associated oxidative stress and inflammatory response^43, 58^. It is well established that these pathways act in a cascading manner. Recent studies suggest, AHR suppresses pulmonary oxidative response and inflammation in response to CC-smoke as a protective mechanism^59, 60^. The role of AHR receptor in maintaining lung Club cell homeostasis have been also reported^61^. As described previously, our multicellular bro-ALI models consist of Club cells following differentiation of the PBEC at ALI^34^. We detected only an increased levels of IL1ꞵ and IL8 in the bro-ALI, whereas in alv-ALI, IFNγ and IL4 levels increased and IL13 decreased following HTP-smoke exposure. However, alteration of secreted levels of other proinflammatory cytokines were not detected. It has been demonstrated that CC-smoke exposure in human bronchial epithelial cell lines induce the release of neutrophil chemoattractant IL1ꞵ and IL8^62^. Alteration of IFNγ, IL4 and IL13 is also consistent with reports demonstrating the differential effects of CC-smoke on the regulation these cytokines in human airway epithelial cells^62, 63^. IL17 pathway is widely implicated in CC-smoke exposure including a positive correlation of IL17 concentration and sputum neutrophil count in COPD patients^64, 65^. Elevated levels of DNA damage response and IL17 have been also reported following CC-smoke exposure^64^. CC-smoke along with other environmental agents activates p38 MAPK pathway in the lung and is involved in pulmonary inflammation in asthma and COPD^62, 66^. However, the physiological relevance of the slight changes in the levels of secreted proinflammatory cytokines detected in this study cannot be ascertained. It is not uncommon to observe a difference between transcript expression profile and secreted levels of cytokines due to the time lag among transcriptional, translational, and secretory processes as also observed in other studies^35, 37^. A time course analysis of the transcript expression and cytokine secretion following HTP-smoke exposure may reveal the kinetics more appropriately.

Dysregulation of estrogen metabolism due to CC-smoke exposure have been detected via upregulation of a key cytochrome P450 gene that can metabolize estrogens such as beta-estradiol to potentially carcinogenic catechol and quinine forms^67^. Glucocorticoid signaling is ubiquitously present in the various organ systems including lung and demonstrate anti-inflammatory and anti-proliferative roles^68^. Alpha-tocopherol is the most bioavailable vitamin E isomer in human tissues and is considered as one of the most potent antioxidant vitamin E which attenuate inflammation specifically via suppressing NFkB mediated cytokine production^69^. LXR is a nuclear hormone receptor with anti-inflammatory properties and are known for triggering “reverse cholesterol transport”^70, 71^. PPARγ represents a disease-relevant pathophysiological and pharmacological target in COPD^72^. PPARγ activation state likely contributes to NF-κB-p65 dependent, CC-smoke induced chemokine-mediated regulation of inflammatory cell accumulation^72^. Exposure to CC-smoke also activates Toll like receptor signaling^58^. NRF2 is a key transcription factor for cell survival during oxidative stress by activating the expression of detoxification and antioxidant genes^58^.

Ferroptosis is a non-apoptotic and oxidative damage-related regulated cell death^73, 74^. Lipid peroxidation plays a central role in mediating ferroptosis^44, 75^. Increased peroxidized phospholipids has been reported in several ferroptosis models^44^. Smoking induced increase in lipid peroxidation can lead to DNA lesions^43^. Elevated levels of MDA have been reported in CC-smoke associated cancers^43^. Measurement of BODIPY-C11 is used to measure lipid peroxidation in ferroptosis^44, 45^. Elevated levels of MDA and BODIPY-C11 were detected in HTP-smoke exposed bro-ALI and alv-ALI. Ferrostatin-1, a synthetic antioxidant, inhibits lipid peroxidation and is also used as a specific inhibitor of ferroptosis^45, 76^. Our findings demonstrate the reduction of lipid peroxidation following ferrostatin-1 treatment in HTP-smoke exposed bro-ALI and alv-ALI models. These findings are consistent with a recent report of CC-smoke induced lipid peroxidation and ferroptosis^77^. This supports the fact that lipid peroxidation and ferroptosis are two important toxicological mechanisms associated HTP-smoke exposure down stream of oxidative stress.

This study provides a broad and comprehensive base to investigate HTP-smoke induced toxicity yet there are some limitations. Regarding the exposure set up, a direct way of exposing the bronchial and alveolar models to HTP-smoke would be preferable compared to the indirect method used in this study. However, due to the operational restrictions of the HTP device, it was difficult to perform direct exposures. In this context, it is also important to note that translation of the dosimetry from our in vitro exposure system to the lung in vivo is difficult. Though the particle number count as well as the particle size distribution is likely to differ between experimental puffing system and real-world puffing and inhalation and deposition across different lung regions yet measurement of the same would have been informative. Possibility to include multiple donors to develop the bro-ALI model for performing the experiments would have been ideal. Moreover, assessment of the HTP-smoke exposure using PBEC from smokers, COPD subjects, asthmatics, and chronic bronchitis patients would have added more dimensions to the study. The NCI-H441 cell line used to develop the alveolar model is an immortalized lung adenocarcinoma cell line which are inherently different compared to normal type II pneumocytes. On the other hand, though primary bronchial epithelial cells were obtained from macroscopically normal regions of the lung in connection with lobectomy, yet the molecular status of those apparently normal lung regions are unknown. Inclusion of immune effector cells such as macrophages in the bro-ALI and alv-ALI would have further added the physiological relevance of the lung mucosa models. This can affect the cell–cell interaction and cross talk that can in turn influence cytokine secretion and release. In this exposure set up, we have restricted ourselves to only one flavour (intense menthol with citrus) of heat-stick. Since flavours corresponds to additives which gives rise to toxic products upon thermal degradation, assessment of multiple flavours together with physical and chemical characterization of HTP-smoke would be necessary in future. Precise measurement of the dose (e.g. total particulate mass) deposited over each transwell membrane will make the exposure methodology more robust. However, similar nicotine concentration was detected in all the wells with low dispersion suggesting comparable exposure dose in all the cell culture plate wells. In this study we have used an ISO puffing regimen and an acute-repeated exposure model. It remains to be seen how the toxicity changes in the event of an intense puffing regimen (e.g. Health Canada Intense)^31^ and chronic exposure settings. The current study mainly focusses on transcriptomic data and corresponding biological pathways. It would be valuable to evaluate the significant pathways at protein level for better functional relevance.

To conclude, we have assessed pulmonary toxicity of HTP-smoke using a broad spectrum of endpoints. This study identified oxidative stress, DNA damage, lipid peroxidation, and ferroptosis as key features of HTP-smoke mediated pulmonary toxicity via a complex regulation of molecular cascade. The findings presented here shows that HTP-smoke exposure is toxic by itself, and the adverse effects are consistent with those reported for CC-smoke. The findings indicate that similar as well as differential toxicological response may drive the effects in different lung regions. Overall, the safety profile of HTP remains questionable. More independent studies are necessary to identify specific mechanisms of HTP use associated toxicity along with studies addressing the long-term adverse effects. This will aid towards evidence-based policy making decisions.

## Supplementary Information

Supplementary Information.

Supplementary Tables.

## Data Availability

All data presented in the study are available on request from the corresponding authors and are also included in the supplementary section. RNA sequencing data is deposited at the Gene Expression Omnibus database at NCBI [GSE198082; use token (ahkpuammjlerlgp) to access].
