# Supplementary material for: Insight into the pulmonary molecular toxicity of heated tobacco products using human bronchial and alveolar mucosa models at air–liquid interface
Source: Sci Rep. 2022 Sep 30;12:16396. doi: 10.1038/s41598-022-20657-y (PMC9525689; doi:10.1038/s41598-022-20657-y)

Supplementary Material

Insight into the pulmonary molecular toxicity of heated tobacco products using human bronchial and alveolar mucosa models at air-liquid interface.

Mizanur Rahman[1], Martin Irmler[2], Micol Introna[1], Johannes Beckers[2,3,4], Lena Palmberg[1],

Gunnar Johanson[1], Swapna Upadhyay[1*#], Koustav Ganguly[1*#]

[1]Unit of Integrative Toxicology, Institute of Environmental Medicine, Karolinska Institutet, Stockholm, 171 77, Sweden

[2]Institute of Experimental Genetics, Helmholtz Zentrum München, Deutsches Forschungszentrum für Gesundheit und Umwelt (GmbH), 85764, Neuherberg, Germany

[3]German Center for Diabetes Research (DZD e.V.), 85764 Neuherberg, Germany

[4]Chair of Experimental Genetics, Technical University of Munich, 85354 Freising, Germany

[#]Equal contribution

[*]**Correspondence**

Koustav Ganguly (koustav.ganguly@ki.se)

Unit of Integrative Toxicology, Institute of Environmental Medicine, Karolinska Institutet, Stockholm, 171 77, Sweden

Swapna Upadhyay (swapna.upadhyay@ki.se)

Unit of Integrative Toxicology, Institute of Environmental Medicine, Karolinska Institutet, Stockholm, 171 77, Sweden

Supplementary table S1 - S6 are provided in the EXCEL file (Supplementary Tables ST1-6)

Supplementary table S1: List of regulated genes ($p<0.01$ and ≥2-fold change compared to sham) in the bro-ALI model 24 h after one day of intermittent exposure to HTP-smoke. 724 genes were upregulated and 300 were down regulated.

Supplementary table S2: Shown are 62 significantly enriched canonical pathways (Fisher`s Exact test p-value < 0.05) associated with the set of 724 up- or down regulated genes in the bro- ALI model 24 h after one day of intermittent exposure to HTP-smoke compared to sham. z-scores > 2 indicate activation, z-scores < -2 indicate inhibition.

Supplementary table S3: 121 genes regulated with $p<0.01$ and ratios >2-fold in alv-ALI post 24 h exposure to HTP-smoke compared to sham. 60 genes were upregulated and 61 were down regulated.

Supplementary table S4: Shown are 21 significantly enriched canonical pathways (Fisher`s Exact test p-value < 0.05) associated with the set of 121 up- or downregulated genes in the alv-ALI model 24 h after one day of intermittent HTP-smoke. z-scores > 2 indicate activation, z-scores < -2 indicate inhibition.

Supplementary table S5: Shown are cytokines with predicted activation state from the IPA Upstream Regulator analysis of the set of 724 regulated genes in bro-ALI model 24 h after one day of intermittent HTP-smoke. z-scores > 2 indicate activation, z-scores < -2 indicate inhibition.

Supplementary table S6: Shown are cytokines with predicted activation state from the IPA Upstream Regulator analysis of the set of 121 regulated genes in alv-ALI model 24 h after one day of intermittent HTP-smoke. z-scores > 2 indicate activation, z-scores < -2 indicate inhibition.

ALI: air-liquid interface; alv-ALI: alveolar mucosa model at ALI; bro-ALI: bronchial mucosa model at ALI; HTP: heated tobacco product; IPA: Ingenuity Pathway Analysis

Nicotine quantification:

Nicotine quantification was performed to inspect its distribution in all wells of the 12 well cell culture plate containing 1mL cell culture medium in each well following 1 puffing session. Nicotine quantification was performed at the Biomedicum Small Molecule Mass Spectrometry Core facility (Karolinska Institutet, Stockholm, Sweden).

Extraction

Samples media was provided in 250 µL Eppenforf tubes. A volume of 25 µL was transferred to a 1.5 mL tube and proteins were precipitated by adding 475 µL of liquid chromatography-mass spectrometry (LC-MS) methanol. Samples were then vortexed and sonicated for 15 minutes on an ice bath (to avoid increases in temperature due to sonication). Next, samples were centrifuged at 10000 g for 15 minutes. Finally, 20 µL were transferred to an LC-MS vial and diluted with 980 µL of LC-MS methanol (50 times dilution).

LC-MS/MS analyses

Samples were analyzed on a ACQUITY UPLC System from Waters Corporation (Milford, MA) coupled to a Waters Xevo TQ-S triple quadrupole system equipped with an electrospray ion source operating in the positive ion mode. The same separation conditions were employed both in positive and negative modes.

Results

Identity of nicotine was confirmed by matching retention time, two SRM transitions and their ion ratio with a nicotine standard (N0267; Sigma Adrich, Saint Louis, MO, USA). A quantification curve was prepared with the nicotine standard and used for quantification. A test was performed to check the signal of nicotine that could fit within the linear range. Dilution was performed in the samples was based on this test. Samples were injected in randomized order. The nicotine concentration of all the 12 wells was 14.1 ± 1.7 µg/mL (mean ± standard deviation) and the percentage of coefficient of variation (%CV) was 11.8. Positive control was injected in triplicate during the sequence as quality control. Results show good reproducibility between the replicates (12.9 ± 0.2; %CV = 1.8). Nicotine was undetectable in all the negative control (cell culture media without HTP-smoke exposure; n=6) samples.

Supplementary Figure S1: Assessment of cytotoxicity using (**a, b**) membrane integrity based propidium iodide (PI) staining by flow cytometry and (**c, d**) colorimetric lactate dehydrogenase (LDH) assay in sham exposed and HTP-smoke exposed bro-ALI and alv-ALI models. Data are shown as percentages of positive PI cells and interquartile ranges. Results from the LDH assay are shown as medians and interquartile ranges. Positive control: kit provided; Negative control: culture media; n = 6 per exposure condition; non-parametric statistical analysis (Wilcoxon signed rank test), *p< 0.05.

ALI: air-liquid interface; alv-ALI: alveolar mucosa model at ALI; bro-ALI: bronchial mucosa model at ALI; HTP: heated tobacco product.

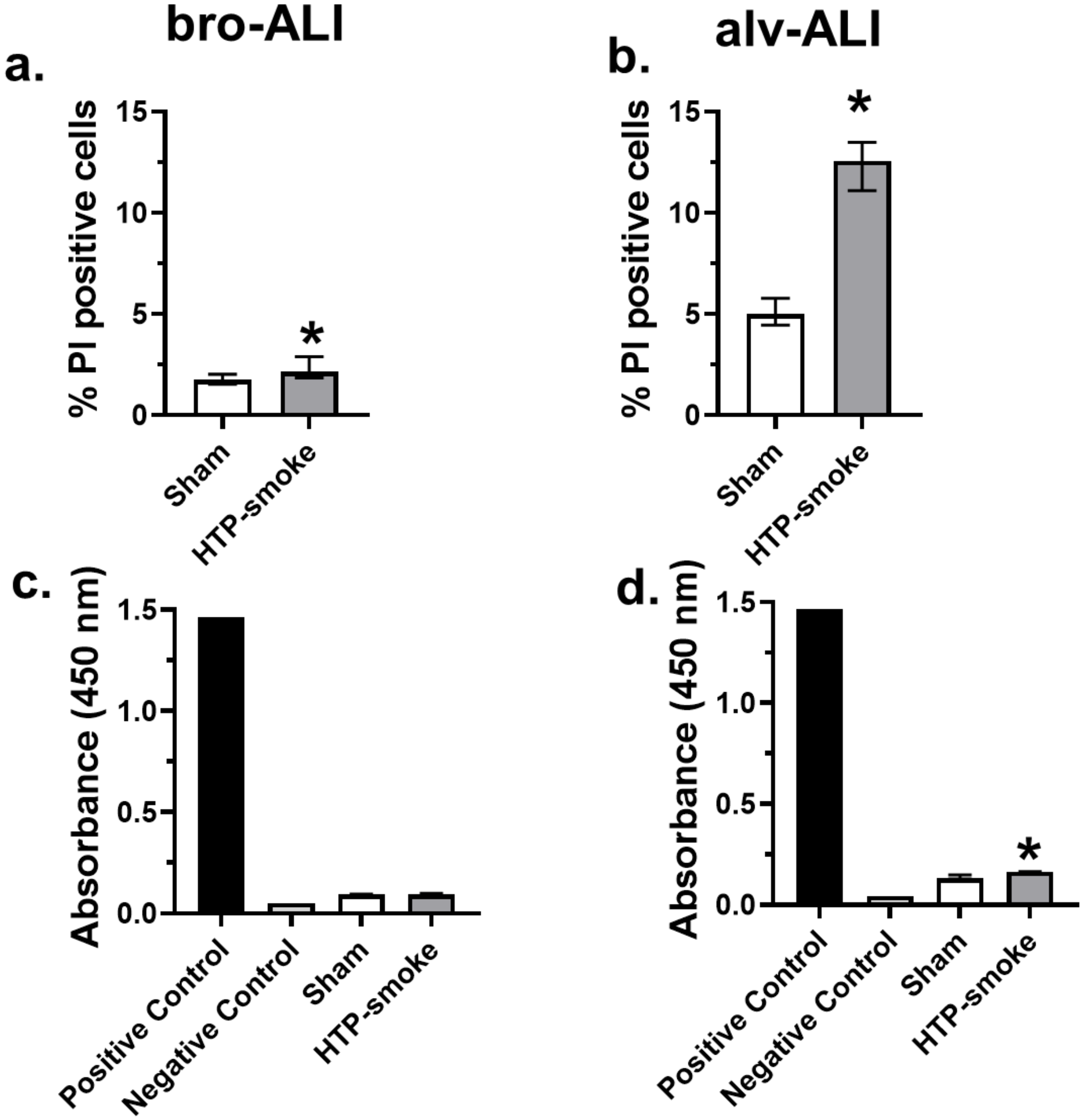

Supplementary Figure S2: Assessment of the transcript expression of selected pro-inflammatory genes in HTP-smoke exposed: (**a**) bronchial (bro-ALI) and (**b**) alveolar (alv-ALI) mucosa models by quantitative real time polymerase chain reaction (qRT-PCR). Beta actin (*ACTB*) was used as the reference control. Fold changes for transcript expression in HTP-smoke exposed bronchial and alveolar models were calculated relative to their corresponding sham. Transcript expression of none of the genes in the HTP-smoke exposed samples were significantly different compared to their corresponding sham. n = 6 per exposure condition; non-parametric statistical analysis (Wilcoxon signed rank test).

ALI: air-liquid interface; alv-ALI: alveolar mucosa model at ALI; bro-ALI: bronchial mucosa model at ALI; HTP: heated tobacco product; IFNG: interferon gamma; IL: interleukin.

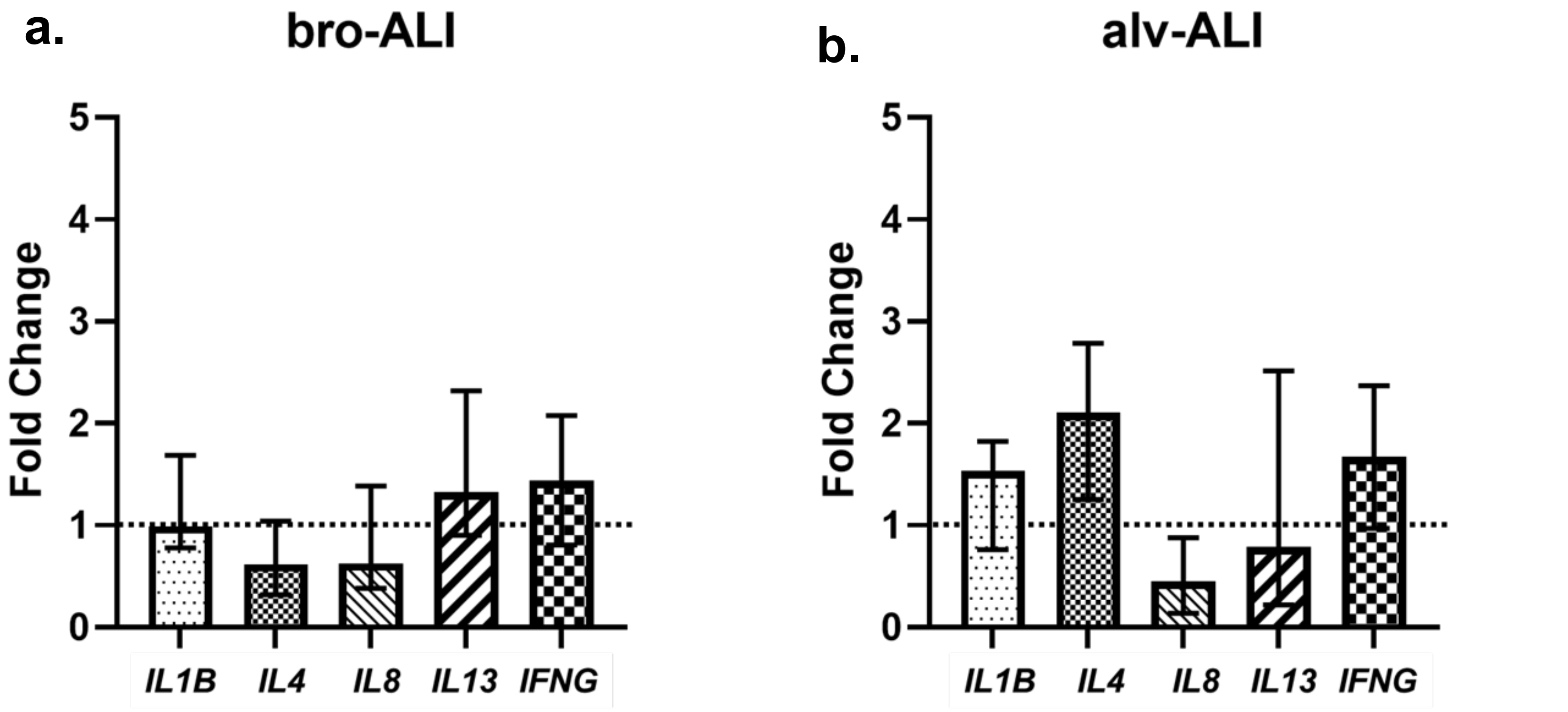

Supplement: Supplementary file 1 — Supplementary Information. [file 41598_2022_20657_MOESM1_ESM.pdf]
